# Three R2R3-MYB transcription factors from banana (*Musa acuminata*) activate structural anthocyanin biosynthesis genes as part of an MBW complex

**DOI:** 10.1186/s13104-023-06375-2

**Published:** 2023-06-13

**Authors:** Mareike Busche, Boas Pucker, Bernd Weisshaar, Ralf Stracke

**Affiliations:** 1 Genetics and Genomics of Plants, Faculty of Biology, Bielefeld University, 33615 Bielefeld, Germany; 2 Institute of Plant Biology & Braunschweig Integrated Centre of Systems Biology (BRICS), TU Braunschweig, 38106 Braunschweig, Germany

**Keywords:** Banana, Flavonoid biosynthesis, *Musa acuminata*, R2R3-MYB, Specialised metabolites, Anthocyanin

## Abstract

**Objective:**

Bananas are one of the most popular fruits in the world, providing food security and employment opportunities in several developing countries. Increasing the anthocyanin content of banana fruit could improve the health-promoting properties. Anthocyanin biosynthesis is largely regulated at the transcriptional level. However, relatively little is known about the transcriptional activation of anthocyanin biosynthesis in banana.

**Results:**

We analysed the regulatory activity of three *Musa acuminata *MYBs that were predicted by bioinformatic analysis to transcriptionally regulate anthocyanin biosynthesis in banana. *MaMYBA1, MaMYBA2* and *MaMYBPA2* did not complement the anthocyanin-deficient phenotype of the *Arabidopsis thaliana pap1/pap2* mutant. However, co-transfection experiments in *A. thaliana* protoplasts showed that *Ma*MYBA1, *Ma*MYBA2 and *Ma*MYBPA2 function as components of a transcription factor complex with a bHLH and WD40 protein, the so called MBW complex, resulting in the activation of the *A. thaliana ANTHOCYANIDIN SYNTHASE* and *DIHYDROFLAVONOL 4-REDUCTASE* promoters. The activation potential of *Ma*MYBA1, *Ma*MYBA2 and *Ma*MYBPA2 was increased when combined with the monocot *Zea mays* bHLH *Zm*R instead of the dicot *At*EGL3. This work paves the path towards decoding the MBW complex-mediated transcriptional activation of anthocyanin biosynthesis in banana. It will also facilitate research towards increased anthocyanin content in banana and other monocot crops.

**Supplementary Information:**

The online version contains supplementary material available at 10.1186/s13104-023-06375-2.

## Introduction

Bananas *(Musa)* are monocotyledonous, perennial plants which are grown in many tropical and subtropical countries. They are one of the most important food crops, particularly in the developing world [1]. While the sweet fruits of dessert bananas are popular in Europe and North America, plantains or cooking bananas are commonly eaten as a staple food in Africa and Latin America where they provide food security, as well as employment opportunities [2]. Furthermore, banana fruits are rich in several health-promoting minerals and beneficial phytochemicals such as vitamins and flavonoids [3].

Flavonoids are a major group of plant specialised metabolites that share a basic structure of two aromatic C6-rings connected by a heterocyclic ring [4]. Reorganisation and modification of the carbon skeleton, such as oxidation, glycosylation, acylation, and methylation create a versatile group comprising more than 9,000 different flavonoid derivatives [5]. Consequently, flavonoids do not only contribute to the nutritional value of fruits, but also play important roles in manifold processes. While the group of coloured anthocyanin pigments attracts animals for pollination and dispersal of seeds by colouring flowers and fruits, other flavonoids protect plants against UV-B irradiation or increase plant fertility [6–10]. Flavonoids from many species have been reported to have anti-pathogenic properties, this includes flavonoids from carnation (*Dianthus caryophyllus*) which have antifungal activity against the plant’s major pest *Fusarium oxysporum* f.sp. *dianthi* [11, 12]. The tropical race 4 (TR4) of the banana Fusarium wilt (commonly known as ‘Panama disease’) is caused by another *Fusarium* subspecies called *Fusarium oxysporum* f. sp. *cubense* (Foc) and threatens the global banana production [13]. Transcriptome analyses of susceptible and resistant banana cultivars infected by Foc TR4 revealed an increased transcription of flavonoid biosynthesis related genes in the resistant cultivar, suggesting an involvement of flavonoids in the defence against Foc TR4 [14].

Flavonoid biosynthesis is one of the best characterised pathways of the specialised metabolism and has been extensively studied in many plant species [15]. In banana, several flavonoid biosynthesis related enzymes have been identified and characterised [16, 17]. Regulation of structural genes on a transcriptional level allows a specific response to environmental influences as well as development and organ specific expression [18–20]. MYB transcription factors are common transcriptional regulators of flavonoid biosynthesis. While some MYBs act independently, others interact with basic helix-loop-helix (bHLH) and WD40 proteins to form a protein complex called MBW complex [21]. MYB proteins are present in all eukaryotes and characterised by highly conserved DNA-binding domains [22]. These MYB domains consist of up to three imperfect amino acid repeat sequences, based on which they are classified. R2R3-MYBs are the most abundant class of plant MYBs and reveal versatile functions in plant-specific processes [23]. Besides core- and specialised metabolism they are also involved in cell fate and -identity definition, developmental processes and the response to biotic and abiotic stresses [23]. Well-known R2R3-MYBs which act as activators of anthocyanin biosynthesis include C1 (COLOURED ALEURONE1) from maize *(Zea mays)*, as well as PAP1 (PRODUCTION OF ANTHOCYANIN PIGMENT1/MYB75) and PAP2 (MYB90) from Arabidopsis *(Arabidopsis thaliana*) [24, 25]. They act as part of an MBW complex and control the promoters of the anthocyanin biosynthesis related structural genes as for example *ANTHOCYANIDIN SYNTHASE* (*ANS*) and *DIHYDROFLAVONOL 4-REDUCTASE* (*DFR*) [26–29].

In banana, 285 R2R3-MYB proteins have been identified in a genome-wide study, including several putative regulators of flavonoid biosynthesis [30]. In addition, MYB31, MYB4 and MYBPR1 – MYBPR4 have been identified as negative regulators of flavonoid biosynthesis in banana [31, 32]. Despite the recent identification of two proanthocyanidin biosynthesis activating R2R3-MYBs [33], little functional data is available on positive regulators (activators) of flavonoid and in particular anthocyanin biosynthesis in *M. acuminata*.

Here, we describe the regulatory properties of three *Ma*MYBs, named *Ma*MYBA1, *Ma*MYBA2 and *Ma*MYBPA2, with a possible role in the regulation of anthocyanin biosynthesis. As one of these *Ma*MYBs was very recently published under the name *Ma*MYBPA2 [33], we used this name to avoid confusion due to multiple protein naming. Regulatory activity was assessed by *in planta* complementation experiments of the anthocyanin deficient *A. thaliana* regulatory mutant *pap1/pap2* and co-transfection experiments in *A. thaliana* protoplasts (see Supplementary File 1 for detailed methods). Our results show that *Ma*MYBA1, *Ma*MYBA2 and *Ma*MYBPA2 are able to activate the promoters of *AtANS* and *AtDFR* as part of an MBW complex. Furthermore, we show that the activation potential of *Ma*MYBA1, *Ma*MYBA2 and *Ma*MYBPA2 is increased when combined with the monocotyledonous bHLH *Zm*R instead of the dicot bHLH protein ENHANCER OF GLABRA3 (*At*EGL3).

## Main text

We aimed to analyse the regulatory properties of three *Ma*MYBs which have been previously assigned to a possible role in positive regulation of anthocyanin biosynthesis (Ma06_g05960 or *MaMYBA1*, Ma09_g27990 or *MaMYBA2*, Ma10_g17650 or *MaMYBPA2*). Since all three *MaMYB* genes were detected in the haploid *M. acuminata* reference genome sequence DH (doubled-haploid) Pahang v2 [34, 35], these *MYBs* appear to be present in the same sub-genome, suggesting that they are different genes and not haplo-copies. We attempted to amplify the corresponding coding sequences (CDSs) on a template collection containing cDNA from different banana samples. The CDSs of all three *MaMYB*s were successfully amplified on cDNA derived from peel tissue of *M. acuminata* (AAA group) cultivar ‘Grand Naine’ grown in the field in Lucknow, India.

**Fig. 1:**
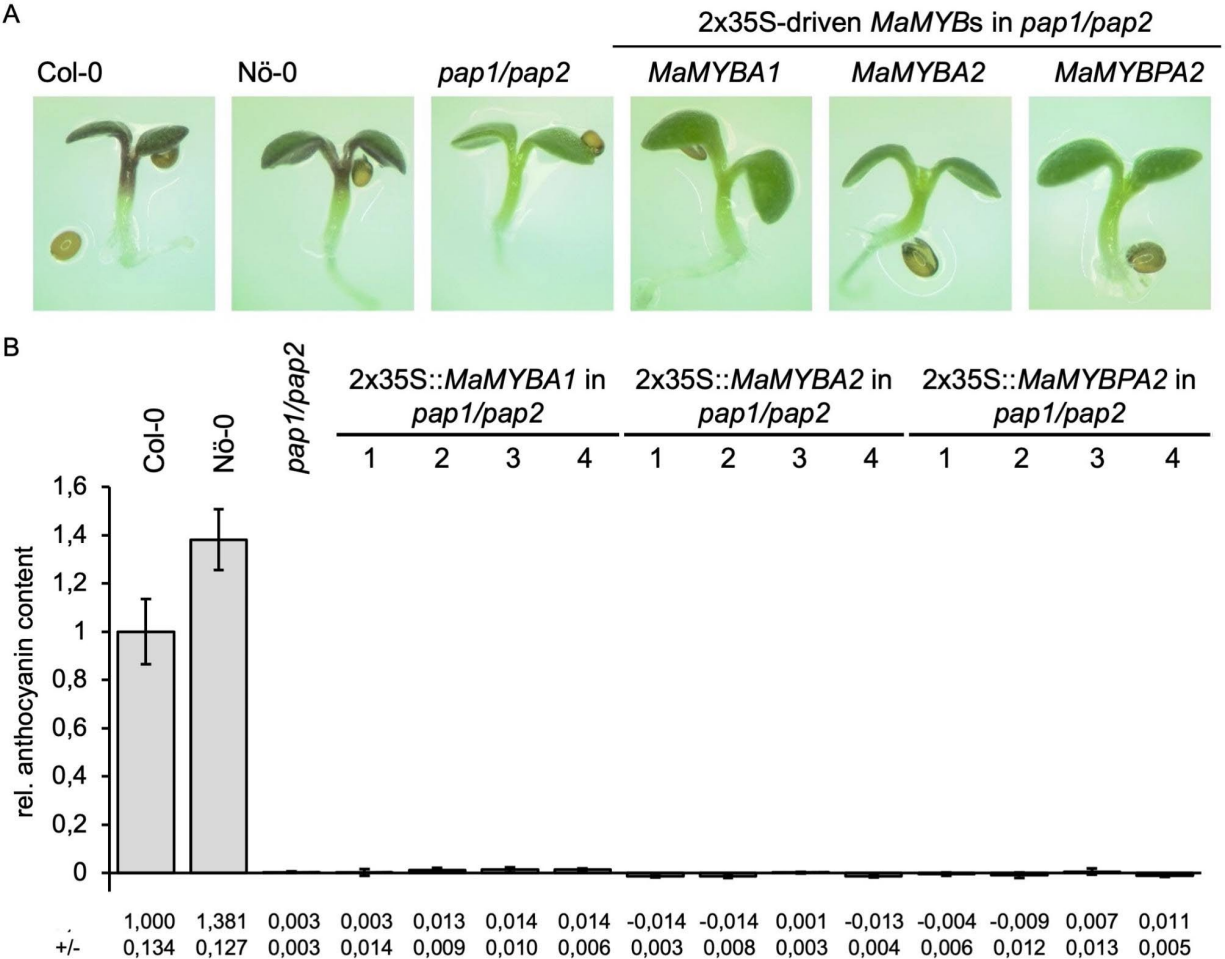
*MaMYBA1, MaMYBA2* and *MaMYBPA2* cannot complement the anthocyanin deficient phenotype of *A. thaliana pap1/pap2* mutant seedlings. **(A)** Representative pictures of anthocyanin accumulation in 6-day-old *MaMYB*-expressing *pap1/pap2* seedlings. One representative plant per construct is shown. **(B)** Photometric measurement of the sucrose induced anthocyanin content in *pap1/pap2* seedlings expressing 2 × 35 S-driven *MaMYB*s. Col-0, Nö-0 (wildtypes) and *pap1/pap2* were used as controls. Error bars indicate the standard deviation of three biological replicates. The different numbers (1-4) represent individual, independent, transgenic lines. The numbers in the table below the graph indicate the relative anthocyanin content and the corresponding standard deviation of individual transgenic lines.

In a first approach, we performed a complementation assay using the regulatory *A. thaliana pap1/pap2* double mutant (*pap1*: transposon tag allele RIKEN_PST16228 in Nö-0 background; *pap2*: T-DNA insertion allele SALK_093731 in Col-0 background [26]), which cannot produce anthocyanins in the seedling (Fig. 1). Seedlings were grown on anthocyanin synthesis-inducing media to analyse the ability of *MaMYBs* under the enhanced cauliflower mosaic virus 35 S promotor (2 × 35 S) to complement the *pap1/pap2* anthocyanin deficiency. While wild-type seedlings (Col-0: Nottingham Arabidopsis Stock Centre (NASC) ID N1092; Nö-0: NASC ID N3081) accumulated high levels of red anthocyanin pigments, *pap1/pap2* seedlings did not. Although *MaMYBA1*, *MaMYBA2* or *MaMYBPA2* were successfully expressed in the transgenic seedlings (Supplementary Figure S1), the anthocyanin level in *pap1/pap2* plants expressing *MaMYBA1*, *MaMYBA2* or *MaMYBPA2* did not differ from that of the double mutant. Accordingly, *MaMYBA1*, *MaMYBA2* and *MaMYBPA2* do not appear to be able to complement the mutant phenotype and thus to regulate anthocyanin biosynthesis in *A. thaliana* in combination with the *bHLH* and *WD40* genes expressed in *A. thaliana* seedlings.

**Fig. 2:**
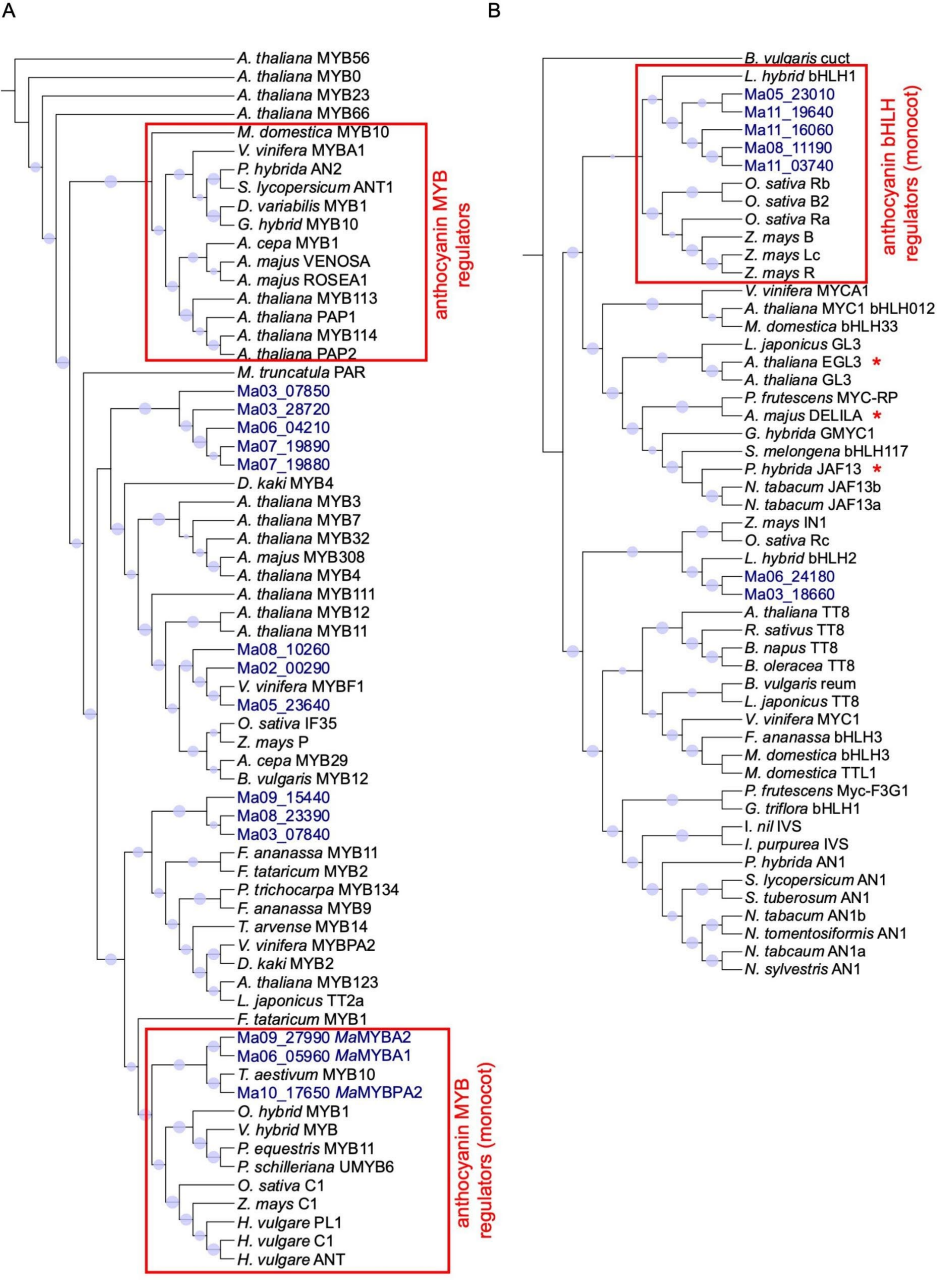
Rooted approximately maximum-likelihood trees of MYB **(A)** and bHLH **(B)** transcription factors. Circle sizes represent bootstrap values. Gene identifiers of banana proteins are highlighted in blue.

Lloyd et al. [28] analysed the *Z. mays* anthocyanin regulator C1 by generating transgenic *A. thaliana* overexpression lines. In their study, Lloyd et al. generated three independent *ZmC1*-expressing *A. thaliana* lines that did not show an increased anthocyanin content compared to wildtype. Similar results were obtained in transgenic tobacco. However, further experiments suggested that *Zm*C1 must interact with the maize bHLH *Zm*R to activate anthocyanin biosynthesis in the heterologous *A. thaliana* system [28]. Phylogenetic analysis [36] showed that the anthocyanin biosynthesis regulating R2R3-MYBs from several monocots such as *Z. mays* and rice (*Oryza sativa*), are part of a different phylogenetic clade than the anthocyanin regulators from several dicots, such as *A. thaliana* or grapevine *(Vitis vinifera)* and further monocots, including onion (*Allium cepa*) and lily (*Lilium hybrida*). Experiments in snapdragon *(Antirrhinum majus)* have also shown that the monocot *Ac*MYB1 can activate anthocyanin production in dicots [36]. The phylogenetic differences between anthocyanin biosynthesis activating R2R3-MYBs and the dependence of *Zm*C1 on *Zm*R in dicots suggest an explanation for our observations. For example, the *Ma*MYBs may depend on their endogenous or at least monocot bHLH for effective activation of structural anthocyanin biosynthesis genes. To investigate the phylogenetic differences between anthocyanin biosynthesis regulating R2R3-MYBs from different plant species, an approximate maximum-likelihood tree was constructed (Fig. 2A). The resulting tree, which also included R2R3-MYBs that activate other branches of flavonoid biosynthesis, revealed two distinct clades of anthocyanin-related R2R3-MYBs (highlighted in red). *Ma*MYBA1, *Ma*MYBA2 and *Ma*MYBPA2 form a clade with MYB10 from *Triticum aestivum* and exhibit a close evolutionary relationship with *Zm*C1, *Os*C1 and anthocyanin regulating MYBs from other monocots. In contrast, *At*PAP1 and *At*PAP2 fall into a second clade of anthocyanin-related MYBs. Furthermore, differences between anthocyanin biosynthesis regulating bHLHs were analysed in a second approximate maximum-likelihood tree (Fig. 2B). The tree showed that monocot bHLH proteins involved in the regulation of anthocyanin biosynthesis form a separate clade (highlighted in red). Both, *Zm*R and several putative anthocyanin biosynthesis regulating bHLHs from banana, fall into this clade. These phylogenetic analyses show that anthocyanin biosynthesis regulating R2R3-MYB and bHLH proteins from several monocot species appear to be distinct from other anthocyanin biosynthesis regulating R2R3-MYB and bHLH proteins, for example from *A. thaliana*. These differences may imply that *Ma*MYBA1, *Ma*MYBA2 and *Ma*MYBPA2 are dependent on a banana or other monocot bHLH.

**Fig. 3:**
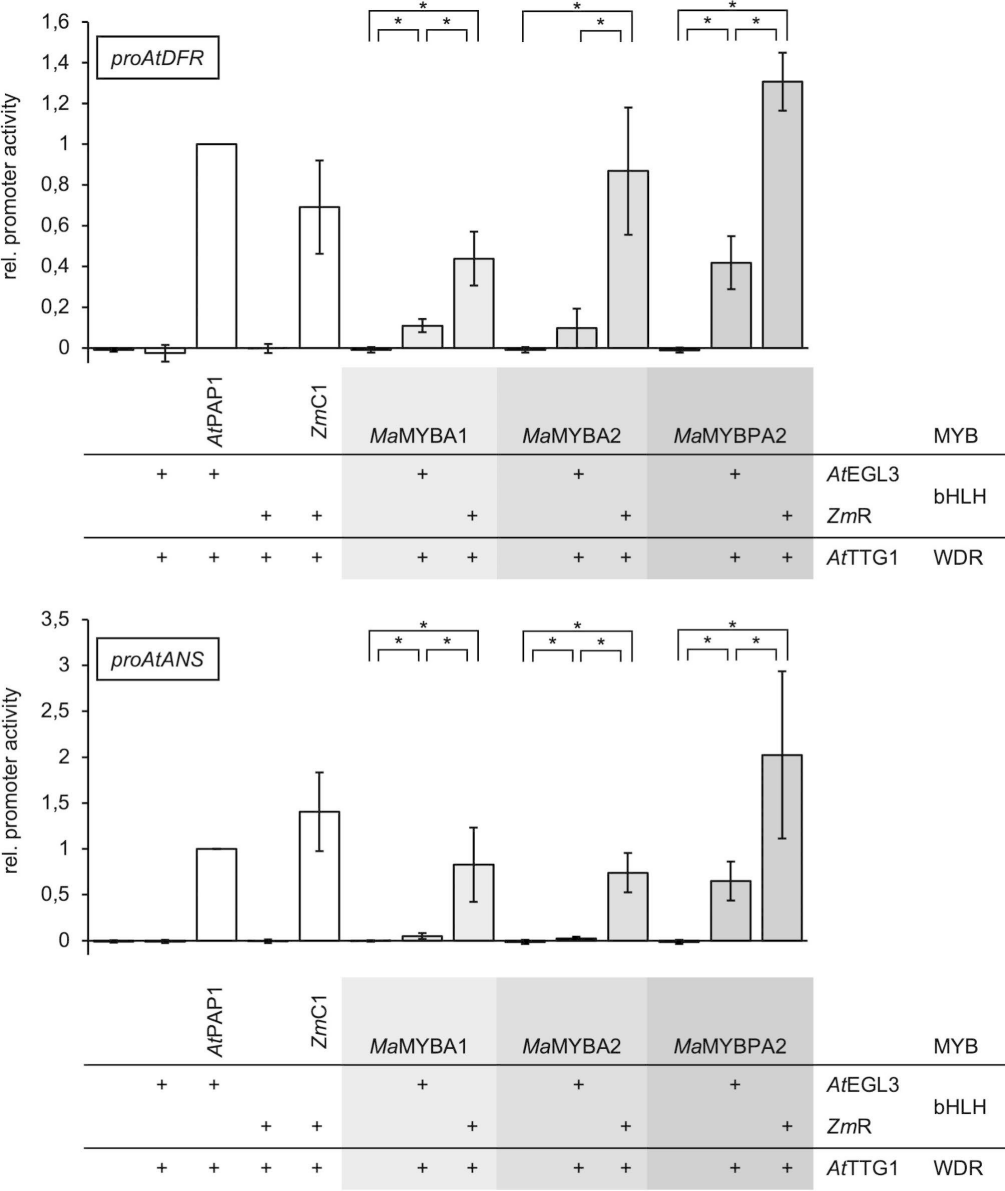
*Ma*MYBA1, *Ma*MYBA2 and *Ma*MYBPA2 can activate *proAtDFR* and *proAtANS* as part of an MBW complex. The ability of *Ma*MYBs to activate *proAtDFR-GUS* and *proAtANS-GUS* reporter constructs in combination with different bHLH proteins (*At*EGL3, *Zm*R) and a WDR (*At*TTG1) was analysed by co-transfection in *A. thaliana* At7 protoplasts. The relative promoter activity refers to the measured GUS reporter enzyme activity. Promoter activity is given relative to the values obtained for the *A. thaliana* MBW complex (*At*PAP1, *At*EGL3, *At*TTG1). Error bars indicate the standard deviation of five independent biological replicates. Statistical significance is indicated by asterisks which mark p-values < 0.05.

To follow up this idea, we further investigated the regulatory properties of the three *Ma*MYBs by performing co-transfection assays (Fig. 3) in hypocotyl-derived, dark-cultured *A. thaliana* At7 protoplasts [37] with different bHLH proteins from *A. thaliana* (*At*EGL3) or *Z. mays* (*Zm*R) and the *A. thaliana* WD40 protein TRANSPARENT TESTA GLABRA1 (*At*TTG1). Their potential to activate the promoters of *AtDFR* and *AtANS*, which are important structural genes of anthocyanin biosynthesis [38], was analysed. While none of the *Ma*MYBs was able to independently activate *proAtANS* or *proAtDFR*, which both contain conserved *cis*-regulatory elements, a slight activation of *proAtDFR* was detected when *Ma*MYBA1 or *Ma*MYBPA2 was combined with *At*EGL3 and *At*TTG1. In combination with *At*EGL3 and *At*TTG1, *Ma*MYBPA2 showed the strongest activation of *proAtDFR* and was also able to activate *proAtANS*. In combination with *Zm*R and *At*TTG1, the three MYBs *Ma*MYBA1, *Ma*MYBA2, and *Ma*MYBPA2 showed a significant activation potential on *proAtDFR* and *proAtANS*. All three *Ma*MYBs showed a higher activation potential in an MBW complex with *Zm*R and *At*TTG1 than in combination with *At*EGL3 and *At*TTG1.

These results show that *Ma*MYBA1, *Ma*MYBA2 and *Ma*MYBPA2 are able to activate *proAtANS* and *proAtDFR* as part of an MBW complex and that *Ma*MYBPA2 shows the strongest activation potential. Furthermore, *Ma*MYBA1, *Ma*MYBA2 and *Ma*MYBPA2 show a higher activation potential when combined with the monocot bHLH *Zm*R instead of the dicot bHLH *At*EGL3.

The phylogenetic differences mentioned above could explain the observed higher activation potentials of the analysed *Ma*MYBs in combination with *Zm*R and *At*TTG1 instead of *At*EGL3 and *At*TTG1. It is conceivable that these differences impede the interaction between the tested *Ma*MYBs and the dicot bHLH *At*EGL3. Since *Ma*MYBPA2 shows the highest activation potential and is also able to activate *proAtANS* in combination with *At*EGL3 or *Zm*R, differences between the three *Ma*MYBs affecting the interaction with the *At*EGL3 are likely and should be further investigated. Interestingly, *Ma*MYBPA2 did not complement the anthocyanin deficient phenotype of *A. thaliana pap1/pap2* mutant seedlings, but was able to activate *proAtANS* and *proAtDFR* when combined with *At*EGL3 in co-transfection experiments in *A. thaliana* protoplasts. Although previous studies have shown *AtEGL3* expression in *A. thaliana* seedlings [21], it is possible that the level was too low to activate anthocyanin biosynthesis in combination with *Ma*MYBPA2. Furthermore, the At7 cell line used for co-transfection experiments was established more than 25 years ago. This long period of propagation in suspension cell culture has caused a number of genomic and transcriptomic changes [39]. These changes may also explain the differences between seedling and cell culture analyses.

The expression profiles of *MaMYBA1*, *MaMYBA2* and *MaMYBPA2* have previously been analysed in embryogenic cell suspension, seedling, root and different developmental stages of leaf, pulp and peel [30]. The expression of *MaMYBA1* was strongest in pulp (developmental stage S2-S3), *MaMYBA2* expression was relatively low in all samples and *MaMYBPA2* showed highest expression in seedlings and early developmental stages of pulp (S1). This gene expression pattern may indicate organ specific *MaMYBA1*, *MaMYBA2* and *MaMYBPA2* activity in banana. Since flavonoid biosynthesis is largely regulated at the transcriptional level, it would be particularly interesting to analyse the expression levels of *MaMYBA1*, *MaMYBA2*, and *MaMYBPA2* in anthocyanin rich organs such as bract or pseudostem to correlate anthocyanin content with *MaMYB* transcript abundance.

Deng et al. [32] performed an expression analysis of genes related to flavonoid biosynthesis using leaves of banana plants overexpressing the anthocyanin repressor *MaMYB4*. They found that the expression of *MaMYBA1* and *MaMYBPA2*, together with *MaDFR* and *MaANS*, is decreased in plants overexpressing *Ma*MYB4, compared to wildtype. These data support a proposed functionality of *Ma*MYBA1 and *Ma*MYBPA2 as transcriptional activators of anthocyanin biosynthesis in banana, as *MaMYB4* could cause a feedback regulation of the positive regulators of anthocyanin biosynthesis, including *MaMYBA1* and *MaMYBPA2*.

Recently, Rajput et al. [33] showed that *Ma*MYBPA2 can activate the banana *ANS, ANR* and *LAR* promoters. They also showed that *Ma*MYBPA2 can partially rescue the proanthocyanin deficiency of the *A. thaliana tt2-1* mutant. The reported ability of *Ma*MYBPA2 to activate *proMaANS* supports the proposed role of *Ma*MYBPA2 in the regulation of anthocyanin biosynthesis. However, the partial complementation of the proanthocyanin deficient phenotype of the *A. thaliana tt2-1* R2R3-MYB mutant, as well as the ability to activate the banana *ANR* and *LAR* promoters, indicates a role in the regulation of proanthocyanidin biosynthesis. Such dual role in the regulation of flavonoid biosynthesis has previously been suggested for R2R3-MYB transcription factors from grapevine, blueberry (*Vaccinium* species) and apple *(Malus domestica)* [40–43]. In several species, including *A. thaliana, Z. mays* and *Petunia hybrida*, anthocyanin biosynthesis is regulated by an MBW complex [21, 28, 44–46]. Our co-transfection assays showed that *Ma*MYBA1, *Ma*MYBA2 and *Ma*MYBPA2 are able to activate *proAtANS* and *proAtDFR* as part of an MBW complex with *Zm*R and *At*TTG1 *in planta*. As shown in a previous study by Pucker et al. [30], it is known that *Ma*MYBA1, *Ma*MYBA2, and *Ma*MYBPA2 are all known to contain a bHLH-binding consensus motif [27]. Thus, the regulation of anthocyanin biosynthesis by *Ma*MYBA1, *Ma*MYBA2, and *Ma*MYBPA2 is likely to depend on bHLH and WD40 proteins. Based on our tree (Fig. 2B), the bHLH encoding genes Ma05_g23010, Ma11_g19640, Ma11_g16060, Ma08_g11190 and Ma11_g03740 could be suitable candidates for further studies on the transcriptional activation of anthocyanin biosynthesis by MBW complexes. In addition, future analyses should additionally include *Ma*TTG1 to identify a complete functional MBW complex in banana and to determine wether the use of endogenous WD40 protein further enhances the activation potential of the MBW complex.

The results presented, in particular the approximate maximum-likelihood trees and the co-transfection assays, show that *Ma*MYBA1, *Ma*MYBA2, and *Ma*MYBPA2 have the ability to transcriptionally activate expression of structural anthocyanin biosynthesis genes in an MBW complex with a suitable bHLH partner. The activation potential of the tested *Ma*MYBs is increased when the *Ma*MYBs are combined with the monocot bHLH *Zm*R instead of the dicot bHLH *At*EGL3.

This is a step towards deciphering the MBW complex-mediated transcriptional activation of flavonoid biosynthesis in banana. It also provides a basis for further research to increase anthocyanin production in banana, which could improve fruit quality and disease resistance.

## Limitations

The co-transfection assays revealed that *Ma*MYBA1, *Ma*MYBA2, and *Ma*MYBPA2 can activate two structural genes of anthocyanin biosynthesis from *A. thaliana* as part of an MBW complex. To further investigate the promotor activation potential of the *Ma*MYBs, the co-transfection analysis could be expanded to other structural genes of anthocyanin biosynthesis, as well as promoters and bHLH and WDR candidates from banana. In addition, yeast two-hybrid or other protein-protein interaction experiments could be performed to investigate the affinity between MYB and bHLH proteins. To elucidate the regulatory role in banana and to confirm possible target genes, future studies should include an overexpression of the three *Ma*MYBs in banana.

## Electronic supplementary material

Below is the link to the electronic supplementary material.

Supplementary Material 1

Supplementary Material 2: Figure S1: Expression analysis of *MaMYBA1, MaMYBA2 and MaMYBPA2* in *A. thaliana pap1/pap2* seedlings. Table S1: Oligonucleotide primers used in this work. Table S2: IDs of protein sequences used for the construction of the phylogenetic tree.

## Data Availability

All data generated or analysed during this study are included in this published article and its supplementary information files.

## Abbreviations

ANS: Anthocyanidin synthase
*A. thaliana*: *Arabidopsis thaliana*
bHLH: Basic helix-loop-helix
C1: Coloured aleurone1
DFR: Dihydroflavonol 4-reductase
EGL3: Enhancer of glabra3
Foc: *Fusarium oxysporum* f. sp. *cubense*
*M. acuminata*: *Musa acuminata*
*O. sativa*: *Oryza sativa*
PAP: Production of anthocyanin pigment
TR4: Tropical race4
TTG1: Transparent testa glabra1
*Z. mays*: *Zea mays*
